# Supplementary material for: Patterns of Intron Gain and Loss in Fungi
Source: PLoS Biol. 2004 Nov 30;2(12):e422. doi: 10.1371/journal.pbio.0020422 (PMC532390; doi:10.1371/journal.pbio.0020422)
Supplement: Table S1 — Also available at http://genes.mit.edu/NielsenEtAl/. (4.3 MB ZIP). [file pbio.0020422.st001.zip › NielsenEtAl/html/662.html]

AN4446.1.NCU04003.1.MG08096.1.FG10067.1


```
 CLUSTAL W (1.82) Multiple Sequence Alignments - Introns Inserted


Sequence 1: NCU04003.1	219 aa
Sequence 2: FG10067.1	219 aa
Sequence 3: MG08096.1	219 aa
Sequence 4: AN4446.1	232 aa
Alignment Length: 233 aa
Number Identitical Residues: 115 aa
Alignment Score (without introns) 5849


MG08096.1 	------------MRPFLPLLTLSTVAQALYFYIDGHAPKCFYEELPKDTLVVG~HYSAEE
NCU04003.1	------------MRSFLPLLSLGAVAQALHFFIDGTSPKCFYEELPKDTLVVG~HYTAEE
FG10067.1 	------------MKFLLPVLSLSAAVNALHFFIDGTTPKCFYEELPKDTLVVG~HYTAEE
AN4446.1  	MAASKRSSWLSSTLAVYAILCFSIPANALYFYVDGRQTRCFYEELPKDTLVVG1TYSSEV
          	 :::. ::  ::   . .:* :.  .:**:*::**  .:**************  *::* 

MG08096.1 	YDDHR--KAWWKHDGISIYISVD0ETFDNDHRVVSQKGSSSGRFTFTAAESGEHKLCFTP
NCU04003.1	WDDQR--HSWQKHDGINIFISVD0ELFDNDHRVVSQRGGPGGKFTFTAAEAGDHRICFTP
FG10067.1 	WDERV--SAWVKHDGISIYINVD0EIFDNDHRVVSQRGLASGRFTFSAADAGDHKICFTP
AN4446.1  	IDQTSGTGTYAVDPSLKMLITVD~EVFDNDHRVVNKRDSHAGRFTFSAADPGNHKICLTP
          	 *:  .: ::  . .:.: *.** * ********.::.  .*:***:**:.*:*::*:**

MG08096.1 	SSNSGNTGWLSTGNQNGGIRLTLDLAIGETSALESSDKDKLEDIATRVKDLNMRLQDIRR
NCU04003.1	TSTSGRSSWMSVNNPNGGIKLTLDMAIGESSAIESADKGKLQDLATRAKDLNNRLQDIRR
FG10067.1 	SSSSGRTSWLSAKNPNGGIKLTLDLVIGETNQIESSDKGKIQDITSRVKDLNARLADIRR
AN4446.1  	ETNAATGGWLSG-APAGAVRVTLDMAIGETSKIESEDKGKISDLVQRVKDLNSRLQDIRR
          	 :.:.  .*:*     *.:::***:.***:. :** **.*:.*:. *.**** ** ****

MG08096.1 	EQVFQR0EREAEFRDQSESTNARVVRWMLIQLTVLGITCAWQLSHLRSFFIKQKLT
NCU04003.1	EQVFQR0EREAEFRDQSESTNARIVRWVLIQLVVLGVTCAWQLSHLRSFFIKQKLT
FG10067.1 	EQVFQR0EREAEFRDQSESTNARVIRWIVIQLIVLGITCTWQLSHLRSFFIKQKLT
AN4446.1  	EQVFQR0EREAEFRDQSEAINSRVVRWTLMQLAVLSVTCAWQLSHLRSFFIKQKLT
          	****** ***********: *:*::** ::** **.:**:****************
```
